# Empathy-Related Brain Activity in Somatosensory Cortex Protects From Tactile Priming Effects: A Pilot Study

**DOI:** 10.3389/fnhum.2020.00142

**Published:** 2020-05-21

**Authors:** Michael Schaefer, Lillia Cherkasskiy, Claudia Denke, Claudia Spies, Hyunjin Song, Sean Malahy, Andreas Heinz, Andreas Ströhle, Michael Schäfer, Nadine Mianroudi, John A. Bargh

**Affiliations:** ^1^MSB Medical School Berlin, Berlin, Germany; ^2^Department of Psychology, Yale University, New Haven, CT, United States; ^3^Department of Anesthesiology and Intensive Care Medicine, Charité – Universitätsmedizin Berlin, Berlin, Germany; ^4^Department of Psychology, Arizona State University, Phoenix, AZ, United States; ^5^School of Humanities and Sciences, Stanford University, Stanford, CA, United States; ^6^Department of Psychiatry and Psychotherapy, Charité – Universitätsmedizin Berlin, Berlin, Germany

**Keywords:** empathy, tactile, priming, social neuroscience, somatosensory cortex, fMRI

## Abstract

Empathy influences how we perceive, understand, and interact with our social environment. Previous studies suggested a network of different brain regions as a neural substrate for empathy, including, in particular, insula and anterior cingulate cortex (ACC). In addition, a contribution of the somatosensory cortices for this empathy related network has been suggested. This is remarkable, given that other recent studies have revealed a role for the somatosensory cortex in various social tasks. For example, in experiments using tactile priming, incidental haptic sensations are found to influence judgment recommendations. Here, we aimed to test if this engagement of the somatosensory cortices during tactile priming can be predicted by the participant’s empathy personality traits. We assessed participant’s empathy and personality traits by means of the Interpersonal Reactivity Index (IRI) and NEO-FFI and tested whether trait empathy is associated with the tactile priming effect in social judgments. Results revealed that empathy predicted the tactile priming effect negatively. This was accompanied by a reduced engagement of the somatosensory cortex, which has been shown to be associated with the priming effect. We conclude that empathy seems to protect people from tactile priming effects.

## Introduction

When we perceive and interact within the social world, empathy seems to be a core component for understanding the emotions and intentions of others. Unfortunately, there is no clear single definition of empathy in current research. Hence, there are different theoretical conceptualizations of this concept. Often (but not always), it is assumed that empathy involves both cognitive as well as affective evaluation processes, thus enabling us to vicariously experience the feelings of another and also understand his or her situation (Hoffman, 2007; Montag et al., 2008; Neumann et al., 2015). Furthermore, empathic feelings have been linked to prosocial behavior (Batson et al., 1981).

Empathy can be seen as multifaceted phenomenon. Therefore, different aspects of empathy depend on different neural substrates. For example, the insula and the anterior cingulate cortex (ACC) seem to be related in particular to empathy for pain (Singer et al., 2004). Beyond this affective network of empathy, recent research also discussed a role for the somatosensory cortices when feeling empathy. In the traditional understanding, the primary somatosensory cortex (SI) reflects touch on the body surface in a more or less mechanical way. Recent research challenged this view. For example, several studies on tactile illusions demonstrate that SI represents the perceived touch rather than the actual touch on the body (Blankenburg et al., 2006; Schaefer et al., 2006, 2007). Moreover, an increasing body of evidence suggests a role for somatosensation in perceiving and understanding social interactions. For example, numerous studies report mirror-like (or vicarious) brain responses in the observer’s SI merely when seeing others being touched, suggesting a putative mirror system in the brain (Keysers et al., 2004, 2010; Blakemore et al., 2005; Schaefer et al., 2009). It has been demonstrated that this vicarious activation in the somatosensory cortices can be linked to empathy personality traits (Gazzola et al., 2006). Thus, the more empathetic an observer is, the more his or her somatosensory cortices are vicariously activated when observing touch on someone else’s body. This has been shown both for painful as well as for non-painful touch [e.g., (Bufalari et al., 2007; Schaefer et al., 2012, 2013)].

While these studies investigated somatosensory-based empathy in very simple or basic experimental settings (e.g., observing a hand or a leg being touched by a paintbrush), few studies report empathy-related responses in the somatosensory cortices in more complex scenarios (Ruby and Decety, 2004; Hooker et al., 2010). However, recent work demonstrates that even complex social-judging processes may engage somatosensory cortices. More concretely, it has been suggested that conceptual (or embodied) metaphors engage in particular sensorimotor brain areas. Conceptual metaphors are different from linguistic metaphors. While the latter are obviously present in language, conceptual metaphors mean understanding and experiencing one kind of thing in terms of another (Lakoff and Johnson, 1980). Several intriguing behavioral studies demonstrate how those embodied metaphors build a scaffold and guide our everyday behavior (Lakoff and Johnson, 1980). Recently, the neural underpinnings of conceptual metaphors have been addressed. Neural substrates of conceptual metaphor effects seem to rely on primary motor and especially somatosensory cortices (Lacey et al., 2012; Schaefer et al., 2014). For example, it has been demonstrated that the moral-purity metaphor is linked predominantly to activity in SI (Schaefer et al., 2015; Denke et al., 2016). Activation in sensorimotor cortices for embodied metaphors is also in line with theoretical assumptions of embodied simulation processes. Simulation here means that the retrieval of conceptual meaning involves a partial re-enactment of sensorimotor experiences (Gallese and Lakoff, 2005).

Given that for both embodied metaphors as well as for empathy personality traits, the somatosensory cortices seem to play important roles, we here hypothesized that conceptual metaphor effects may be affected by empathy. Thus, behavioral effects and also neural activation during embodiment effects may be predicted by empathic personality traits.

In order to test our hypothesis, we reanalyzed data from our previous fMRI study (Schaefer et al., 2018, Study 2), in which we investigated tactile priming effects in a social judging paradigm (“hardness” metaphor). Previous research investigated the psychological concept of “hardness” and suggested that it may be an example for a conceptual metaphor. Thus, activation of the “hardness” concept through actual physical experiences might guide analogous psychological concepts. What kind of psychological concepts are associated with “hardness”? “Hardness” can be related to idioms such as “hard-hearted” or having a “hard day,” pointing in particular to the metaphorical meaning of resistance to outside influence. Recent studies by Ackerman et al. (2010) demonstrate how the physical experience of “hardness” affects social behavior (see also Xie et al., 2016). In their experiment, participants were asked to imagine buying a new car. In this role-playing scenario, they first made an offer to the dealer, who rejected this offer, asking for a second offer. Participants sitting in a hard wooden chair (in contrast to a soft cushioned chair) made a smaller adjustment to their first offer. Thus, they did not compromise as much as did those seated on soft chairs. In other words, they took a “harder line” in their negotiations (Ackerman et al., 2010).

Our previous research sought to extend this research by testing whether incidental “hard” sensations lead people to be “hard” on crimes and by investigating the underlying neural substrates of this conceptual metaphor. Participants were asked to recommend punishments in the context of crime scenarios while lying in the scanner. Before reading each crime scenario they were primed with either hard or soft tactile stimuli (or no tactile stimulation) by an experimenter next to the participant (participants touched either a hard or a soft surface for few seconds). In line with a “hard on crime” conceptual metaphor, we demonstrated that hard-priming led to harder punishments (relative to soft and relative to no tactile stimulation) and that this effect is based in particular on SI (no effects for comparison soft relative to no stimulation). Based on these findings, we hypothesize here that both the behavioral priming effect as well as the neural activation in SI (but not activation in other brain regions) can be predicted by empathy personality dimensions (but not by other personality dimensions). In order to test this hypothesis, we assessed empathy and Big-Five personality traits and examined whether empathy personality traits moderated the behavioral priming effect and the associated activation in SI.

## Materials and Methods

### Participants

Fifteen out of the 17 participants that participated in the previous study (Schaefer et al., 2018) were included in the current analyses (11 females, mean age 23 ± 2.84 years).

All participants were native German volunteers with no neurological or psychiatric history. The participants gave written informed consent to the study, which adhered to the Declaration of Helsinki and was approved by the by the local human subjects committee. The data that support the findings of this study are available on request from the corresponding author (MS).

### Procedure

Participants were asked to complete two personality questionnaires, a German version of the NEO Five-Factor Inventory (NEO-FFI) (Costa and McCrae, 1992; Borkenau and Ostendorf, 1993) and a German version of the Interpersonal Reactivity Index (IRI) (Davis, 1983; Paulus, 2009).

The IRI has been previously used in imaging studies to examine empathy-related brain activations (e.g., Singer et al., 2004; Bufalari et al., 2007). It is a 28-item survey that consists of four subscales (Davis, 1983; Paulus, 2009), resulting in an affective (or emotional) and a cognitive form of empathy. The scale “perspective taking” (PT) represents the tendency to cognitively imagine a situation from the other person’s point of view. A subscale “fantasy” (FS) assesses the tendency to project oneself into the place of fictional characters in books or movies. The scale “empathic concern” (EC) measures a person’s tendency to have feelings of sympathy and concern for others. A fourth scale, the “personal distress” scale (PD), reflects the extent to which someone feels negative emotions, especially in stressful situations. According to Davis, EC, and PD describe an affective form of empathy, whereas the subscales PT and FS assess cognitive empathy (Davis, 1983).

The NEO-FFI is based on a factor-analytic approach describing the human personality in five core dimensions, which are extraversion, neuroticism, agreeableness, conscientiousness, and openness to experience (Costa and McCrae, 1992). Neuroticism involves experiencing negative emotions (including anxiety), self-consciousness, and irritability. Extraversion is linked toward a tendency to experience positive emotions, including a high degree of sociability, assertiveness, and talkativeness. Agreeableness is displayed by a tendency to altruism, including traits such as cooperation, compassion, and politeness. Conscientiousness is linked to disciplined, organized, and achievement-oriented behavior. Openness to experience is reflected by active imagination, aesthetic sensitivity, attentiveness to inner feelings, preference for variety, and intellectual curiosity (Costa and McCrae, 1992). The NEO-FFI includes 60 items and is widely used to examine Big-Five personality traits.

The fMRI study design included one factor, tactile priming, which was hard, soft, or omitted (no tactile stimulation). Tactile stimulation was carried out via foam (soft stimulation) or wood material (hard stimulation). The stimuli were comparable with respect to weight, size, and shape. The experimenter held the object in one hand and let the participants touch the hardness of the stimuli. The participants were made familiar with this task outside the scanner. Participants were not able to see the priming objects or to freely explore their shape or weight or swipe on the object’s surfaces. That is, participants were only allowed to press and feel the hardness or softness of the objects by using thumb and residual fingers while this object was held by the experimenter. It is important to note that we never talked about “hardness” when instructing the participants. The participants were merely told that they were sometimes going to feel objects during the experiment. We did not give participants any additional information on this task.

When lying in the scanner, participants were primed either with hard wood or soft foam for about 15 s (hard, soft, and no tactile priming) before they read scenarios describing crimes such as burglaries, criminal assaults, murderers, cheating, or drug offenses (16 s each). All scenarios included both positive (mitigating) and negative components to ensure that they were ambivalently valenced. For example, subjects read the following scenario: “A 20-year-old man drove his friends to a night club. Because he was intoxicated and the atmosphere in the car was distracting, he ran a red light, thereby causing a serious traffic accident. Later on, the young driver apologized for his actions when meeting the injured persons.” Then, participants gave their judgment as to how seriously the protagonist should be punished (8-point Likert-scale) (“How seriously should the young man be punished? More seriously: right buttons. Less seriously: left buttons”). After 14 s, there was a break of 12 s before the next trial started.

The visual images were back-projected to a screen at the end of the scanner bed, close to the subject’s feet. Participants viewed the scenarios through a mirror mounted on the birdcage of the receiving coil. The experiment consisted of a total of 60 scenarios (four runs, each lasting about 14 min). The order of presentation of the scenarios as well as the kind of priming for the scenarios was randomized between and within the subjects. Conditions were randomized within runs. Participants were familiarized with the task before starting the experiment. We used a cover story for the participants by telling them that they would participate in two separate and independent experiments in one session: The first experiment referred to the examination of neural correlates for touch stimuli, while the second experiment examines neural correlates of judgment processes. At the end of the study all participants were debriefed and probed for suspicions concerning the experiments. For further details with respect to the experimental procedures, see Schaefer et al. (2018).

### fMRI Data Acquisition and Analysis

The functional imaging was conducted by using a 3T scanner (Siemens MAGNETOM Trio, Germany). T2-weighted functional images were acquired using gradient echo-planar images (TR = 2 s, TE = 30 ms, flip angle = 80 degrees, FOV = 192 mm). For each participant, data were acquired in four runs. Each run consisted of 404 volumes. Functional volumes included 32 slices (3.5-mm slices, no gap, in plane voxel size 3.5 × 3.5 mm). A high-resolution, T1-weighted structural image was acquired for anatomic reference (MP-RAGE, TR = 1650 ms, TE = 5 ms).

Individual functional images were realigned to correct for inter-scan movement using sinc interpolation and were subsequently normalized into a standard anatomical space (MNI, Montreal Neurological Institute template), resulting in isotropic 3-mm voxels. Data were smoothed with a Gaussian kernel of 6 mm, full-width, half-maximum. Data preprocessing and statistical analyses were carried out using the Statistical Parametric Mapping Software (SPM, Wellcome Department of Imaging Neuroscience, University College London, London, United Kingdom).

Statistical parametric maps were computed using multiple regression with the hemodynamic response function modeled in SPM. We examined brain responses while participants gave the punishment recommendations and then calculated statistical contrasts (*t*-tests) with respect to the different priming conditions. In order to test our hypothesis, we tested if there are linear relationships between personality traits (empathy, Big Five) and the size of the tactile priming effect (behavioral effect and brain responses). Therefore, scores of the personality traits (IRI, NEO-FFI) were used to test for correlations (Pearson) with brain activation (parameter estimates for voxels) in the sensorimotor regions of interest (maximum peaks in bilateral SI) and other regions (premotor cortex, inferior frontal gyrus, and secondary somatosensory cortex; separately analyzed and FWE corrected) (see Table 1 and Schaefer et al., 2018).

**TABLE 1:** Behavioral and fMRI results for “hard on crime” effect.

Priming condition	Behavioral results (punishment recommendations, scale from 1 to 8, with 8 for very hard sentences, means ± standard deviation)	Brain region	Peak MNI location (*x, y, z*)	Peak *z*-value	Number of voxels
Hard > soft priming	Hard priming: 4.74 ± 0.98 Soft priming: 4.33 ± 0.65	R SI	44, −40, 66	3.80	32
Hard > no priming	Hard priming: 4.74 ± 0.98 No priming: 4.30 ± 0.55	(L SI) (L BA6) (R BA6) (R SII/Insula) (L SII/Insula) (R inf. frontal gyrus/BA44)	−24, −26, 50 −32, −4, 38 22, −16, 58 40, −12, 2 −28, 8, 16 62, 12, 6	3.50 3.70 3.39 3.46 3.28 3.35	19 8 12 31 7 6

*Results demonstrate significant “harder” decisions after hard priming [ANOVA with factor priming condition, *F*(2,28) = 3.73, *p* = 0.03], accompanied with activation in somatosensory and other brain areas [random effects analysis, *p* < 0.05, FWE corrected, L, left hemisphere; R, right hemisphere; in brackets, uncorrected results, see Schaefer et al. (2018) for further details].*

Traits that showed linear relationships went into standard multiple linear regression analyses to analyze the relationships between the “hard on crime” effect (and the associated brain activity) and personality traits. The “hard on crime” effect is expressed in comparisons of hard relative to no priming and hard relative to soft priming (see Table 1). The software package SPSS (IBM Corp., Armonk, NY, United States) was used for all statistical analysis.

## Results

### Questionnaire and Behavioral Data

Mean scores of the IRI questionnaire are depicted in Table 2. FS correlated with EC (*r* = 0.51, *p* = 0.05, Pearson, two-sided). Table 2 also depicts results for NEO-FFI. Neuroticism correlated significantly with agreeableness (*r* = −0.61, *p* = 0.02). There were no other significant correlations between personality or IRI dimensions.

**TABLE 2:** Results of personality questionnaires (IRI and NEO-FFI).

Personality questionnaires		Mean ± standard deviation
IRI	*Perspective taking*	14.80 ± 1.97
	*Fantasy*	14.73 ± 2.69
	*Personal distress*	9.80 ± 1.90
	*Empathic concern*	15.27 ± 1.67
NEO-FFI	*Neuroticism*	16.86 ± 7.55
	*Extraversion*	29.29 ± 7.52
	*Openness to experience*	34.50 ± 5.87
	*Agreeableness*	31.71 ± 4.94
	*Conscientiousness*	34.00 ± 6.23

We first tested if personality affected punishment recommendations in general, irrespective of priming. Results revealed that in particular EC affected general punishment behavior. Both EC and PD correlated negatively with the severity of punishment recommendations (EC: *r* = −0.53, *p* = 0.04; PD: −0.43, *p* = 0.10). A subsequent regression analysis with the four empathy dimensions as predictors confirmed this result [*R* = 0.61, adj. *R*^2^ = 0.45, *F*(4,14) = 3.92, *p* = 0.03]. The IRI dimension EC predicted the general severity of recommendations (β = −0.77, *p* < 0.01). Hence, the more empathic the participant was, the more lenient was his or her punishment, as expected. Other empathy dimensions (PD, PT, F, or Big-Five personality traits) did not show any significant effects on general punishment recommendations (all *p* > 0.10) (see Table 3).

**TABLE 3:** Intercorrelation matrix for empathy measures, behavioral results, and brain activation (Pearsons, in bold: *p* < 0.05).

		Empathy (IRI)				Behavioral responses			Brain activationh	
		EC	PD	PT	FS	General severity of punish. recom.	Diff. of punishment recom. after hard – no tactile priming	Diff. of punishment recom. after hard – soft tactile priming	Diff. of brain activation in SI after hard – no tactile priming	Diff. of brain activation in SI after hard – soft tactile priming
Empathy (IRI)	EC		*r*=0.17, *p*=0.53	*r*=0.23, *p*=0.40	*r*=0.51, *p*=0.05	***r* =** −**0.53**, *p* = **0.04**	***r* =** −**0.60**, *p* = **0.01**	*r* = −40, *p* = 0.13	***r* =** −**0.55**, *p* = **0.03**	*r* = −0.47, *p* = 0.07
	PD			*r*=−0.33, *p* = 0.22	*r*=0.21, *p*=0.44	*r* = −0.43, *p* = 0.10	*r* = −0.24, *p* = 0.37	*r* = −43, *p* = 0.10	*r* = −0.13, *p* = 0.63	*r* = −0.45, *p* = 0.08
	PT				*r*=0.03, *p*=0.91	*r* = 0.26, *p* = 0.33	*r* = 0.20, *p* = 0.47	*r* = 0.20, *p* = 0.46	*r* = 0.31, *p* = 0.25	*r* = −0.12, *p* = 0.65
	FS					*r* = −0.02, *p* = 0.92	*r* = −0.41, *p* = 0.12	*r* = −0.28, *p* = 0.30	*r* = −0.17, *p* = 0.53	*r* = −0.38, *p* = 0.16
Behav. responses	Gen. sev.						***r* = 0.57**, ***p* = 0.02**	*r* = 0.45, *p* = 0.08	***r* = 0.53,** ***p* = 0.04**	*r* = 0.34, *p* = 0.20
	Hard – no							***r* = 0.77,** ***p* = 0.01**	*r* = 0.48, *p* = 0.06	***r* = 0.67,** ***p* = 0.01**
	Hard – soft								*r* = 0.48, *p* = 0.06	***r* = 0.56,** ***p* = 0.02**
Brain activation	Hard – no									*r* = 0.24, *p* = 0.37
	Hard – soft									

**FIGURE 1:**
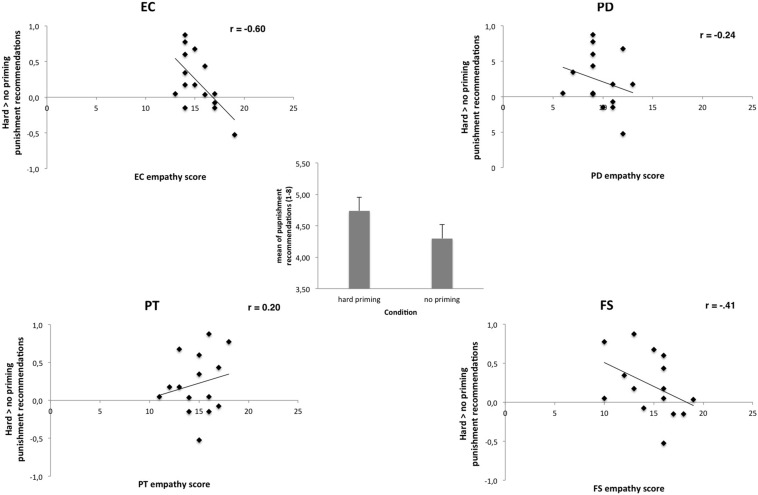
Correlation scatterplots for empathy scores of IRI with punishment recommendations after hard vs. no priming. Only the IRI dimension EC predicted negatively the strengths of the “hard on crime” effect (Pearson, EC: *r* = −0.60, *p* = 0.01; PD: *r* = −0.24, *p* > 0.10; PT: *r* = 0.20, *p* > 0.10; FS: *r* = −0.41, *p* > 0.10). Center shows mean punishment recommendations after hard and after no priming. See text for further details.

We then tested whether tactile priming (the “hard on crime” effect) depends on the personality of the participants. The “hard on crime” effect describes that tactile priming with hard (relative to soft or relative to no priming) surfaces makes our punishment recommendations to be “harder” (Schaefer et al., 2018). Examining linear relationships between personality measures and this tactile priming effect revealed that, in particular, EC correlated in a linear fashion with the strength of the “hard on crime” effect (hard relative to no priming, EC: *r* = −0.60, *p* = 0.01; hard relative to soft priming, EC: *r* = −0.40, *p* = 0.13; PD: *r* = −0.43, *p* = 0.10). Other empathy dimensions failed to show linear relationships (see Figure 1).

We then calculated a regression analysis with all four IRI empathy dimensions as predictors [all four scales were entered simultaneously in one model; hard relative to no priming; *R* = 0.70, adj. *R*^2^ = 0.30, *F*(4,14) = 2.51, *p* = 0.10]. Results demonstrated that in particular EC predicted the “hard on crime” effect (EC: β = −0.64, *p* = 0.04; PD: β < 0.01, *p* = 0.97; PT: β = 0.35, *p* = 0.18; FS: β = −0.10, *p* = 0.70). The contrast hard relative to soft priming showed failed to reach the level of significance [*R* = 0.57, adj. *R*^2^ = 0.06, *F*(4,14) = 1.24, *p* = 0.35; EC: β = −0.37, *p* = 0.26; PD: β = −0.30, *p* = 0.32, PT: β = 0.19, *p* = 0.51; FS: β = −0.03, *p* = 0.92] (see Table 4).

**TABLE 4:** Regression analyses of behavioral results (difference of punishment recommendations for hard relative to soft and for hard relative to no priming) with empathy subscales as predictors.

	Model					Coefficients (standardized)		
	*R*	*R*^2^	Adj. *R*^2^	ANOVA		Betas	*T*	Sign.
Hard relative to no priming	0.70	0.50	0.30	*F*(4,14) = 2.51, *p* = 0.10	EC: PD: PT: FS:	−0.64 0.01 0.35 −0.10	−2.33 0.03 1.42 −0.39	*p* = 0.04 *p* = 0.97 *p* = 0.18 *p* = 0.70
Hard relative to soft priming	0.57	0.33	0.06	*F*(4,14) = 1.24, *p* = 0.35	EC: PD: PT: FS:	−0.37 −0.30 0.19 −0.03	−1.19 −1.04 0.67 −0.10	*p* = 0.26 *p* = 0.32 *p* = 0.51 *p* = 0.92

*All four IRI dimensions (EC, F, PT, and PD) went simultaneously in one model.*

Thus, in particular, EC seems to negatively influence the strength of the behavioral shown “hard on crime” effect. The more empathetic the participants were, the weaker was the influence of the “hard on crime” effect. However, it has to be noted that (perhaps due to small sample sizes) both regression models did not reach the level of significance. In addition, the contrast “hard–soft” revealed no significant empathy predictors at all.

**FIGURE 2:**
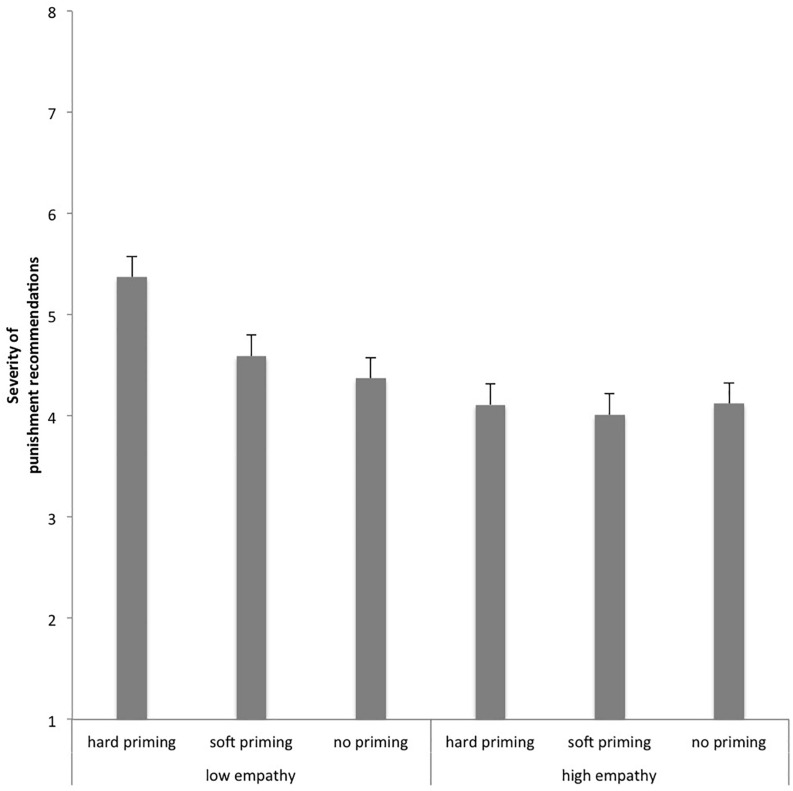
Behavioral results split for participants with high and low empathy scores (global empathy score including EC, PT, and FS).

Figure 2 splits the “hard on crime” effect for participants with high and low empathy scores (participants with highest vs. lowest global empathy scores, including EC, PT, and FS) and suggests that, in particular, participants with low empathy scores seem to be prone for the “hard on crime” effect.

### fMRI Data

Our previous study demonstrated that brain responses underlying the “hard on crime” effect engaged predominantly the somatosensory cortex. Thus, brain activation during judging when being primed with the hard object compared with soft priming (and with no priming) revealed activation in somatosensory cortex on the right hemisphere. Results for the left-side revealed similar activation in somatosensory cortex at a lower level that did not surpass correction for multiple comparisons (see Table 1 and Schaefer et al., 2018 for details). We concluded that the “hard on crime” effect seems to be linked to an activation of the somatosensory cortices.

**FIGURE 3:**
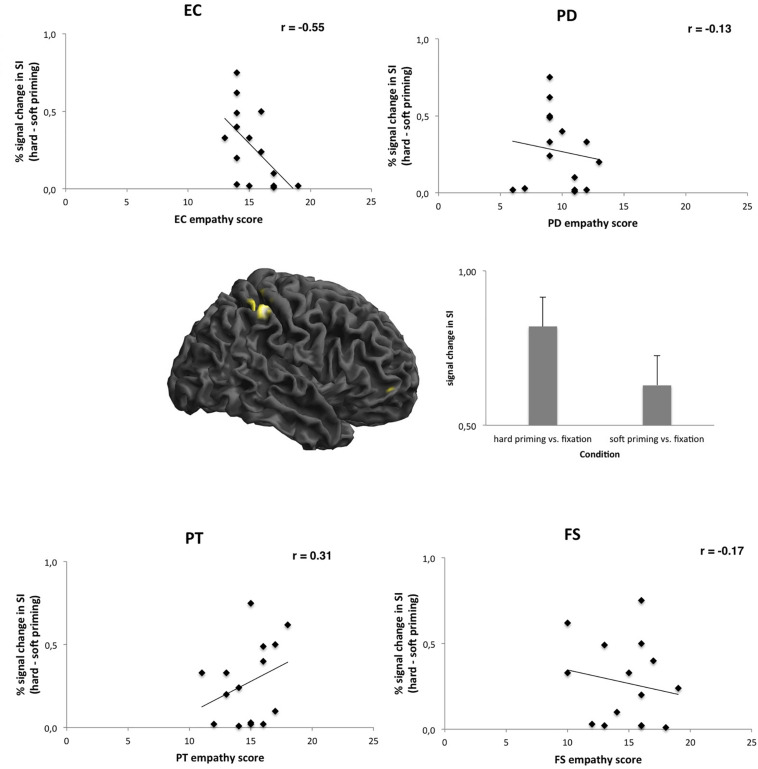
Brain activation in right somatosensory cortex after hard priming (relative to soft priming) (peak MNI locations *x* = 44, *y* = −40, *z* = 66; *p* < 0.05, FWE corrected). Correlation scatterplots for signal change in this brain area with empathy scores demonstrate linear correlations with EC empathy (Pearson, EC: *r* = −0.55, *p* = 0.03; PD: *r* = −0.13, *p* > 0.10; PT: *r* = 0.31, *p* > 0.10; FS: *r* = −0.17, *p* > 0.10). Bar graph in center shows average BOLD signal changes relative to rest (fixation) condition.

The present study aimed to examine whether personality traits are linked to the neural activation in SI (during the punishment recommendations) associated with the “hard on crime” effect. In order to test this hypothesis, we calculated a regression analysis for brain changes in SI related to the “hard on crime” effect with EC as a predictor. Results demonstrated that empathy had an influence on the neural activity in SI [hard relative to soft priming; *R* = 0.55, adj. *R*^2^ = 0.25, *F*(1,14) = 5.81, *p* = 0.03], demonstrating that EC was a predictor for the “hard on crime” effect (β = −0.55, *p* = 0.03, see Figure 3 and Table 5). An analog regression analysis for brain changes in SI for the contrast hard relative to no priming including EC as a predictor confirmed these results, but showed only a trend for significance [*R* = 0.47, adj. *R*^2^ = 0.16, *F*(1,14) = 3.68, *p* = 0.07]. The predictor EC showed a negative effect for the “hard on crime” effect (β = −0.47, *p* = 0.07). No other personality traits (Big Five) revealed linear relationships with brain activation in SI (all *p* > 0.10).

**TABLE 5:** Regression analyses of fMRI results (difference of brain activation in SI for hard relative to soft and for hard relative to no priming) with empathy subscale EC as predictor (based on behavioral analyses; only EC went into the model).

	Model					Coefficients (standardized)		
	*R*	*R*^2^	Adj. *R*^2^	ANOVA		Betas	*T*	Sign.
Hard relative to no priming	0.47	0.22	0.16	*F*(1,14) = 3.68, *p* = 0.07	EC:	−0.47	−1.91	*p* = 0.07
Hard relative to soft priming	0.55	0.30	0.25	*F*(1,14) = 5.81, *p* = 0.03	EC:	−0.55	−2.41	*p* = 0.03

These results demonstrated that empathy personality traits were linked in a negative way to somatosensory activity in a tactile priming task. Thus, the more empathic participants were EC, the weaker was the “hard on crime” effect related signal changes in SI.

Similar calculations for signal changes in other brain areas (premotor cortex, inferior frontal gyrus, secondary somatosensory cortex) revealed no significant relationships with empathy or Big Five personality measures.

## Discussion

Based on recent findings that discussed a role for the somatosensory cortices both in empathy as well as in tactile priming scenarios, the present pilot study aimed to test whether empathic personality traits may interact with tactile priming effects in social judgments. We here demonstrated that the degree of EC could predict the magnitude of the priming effect in the participant’s judgments and the associated somatosensory activation, thereby suggesting that empathy may protect from priming effects in the social sphere.

Our results revealed that both the behavioral as well as the neuroimaging data show correlations with empathic personality traits. On a behavioral level we found that the tactile priming of social judgments correlated with EC (although, comparison with soft priming failed to reach the level significance). The more empathic the participants were, the less strong was the priming effect. Imaging results confirmed these results. The more emotionally empathic the participants were, the less was the somatosensory cortex (here, the underlying neural substrate of this tactile priming effect) engaged. Hence, empathy seems to be linked to tactile priming effects in social judgments.

Numerous studies report an engagement of the somatosensory cortices for empathy (Preston and De Waal, 2002; Gallese and Lakoff, 2005; Preston, 2013; De Waal and Preston, 2017). For example, prior studies investigating the general relationship between trait empathy (measured with the IRI) and neural activity found individual differences in gray matter volume in the precuneus, anterior cingulate, somatosensory cortex, and insula for EC and PD. For PT and FS anterior cingulate and dorsolateral prefrontal cortex seem to be important (Banissy et al., 2012). In addition, a lack of empathy has been related to altered somatosensory functioning (e.g., Khan et al., 2015). Although an increasing body of evidence demonstrate a role for somatosensory cortex, the exact contribution of this region for empathy [and its interplay with other brain areas known to be related to empathy, e.g., the insula and ACC (Singer et al., 2004)] still remains to be cleared (Gazzola et al., 2006; Schaefer et al., 2012; Allen et al., 2017; Gallo et al., 2018). Several studies showed that tactile priming of social judgments engages the somatosensory cortices, too (Schaefer et al., 2014, 2018; Denke et al., 2016). In addition, it has been revealed that the neural underpinnings of a general understanding textual metaphors involve somatosensory brain areas (Lacey et al., 2012). The present study confirms and extends these results by demonstrating a relationship between empathy and priming related somatosensory activation. However, the involvement of the somatosensory cortex in our study seems to be predominantly linked to tactile priming effects. In other words, the more participants are prone to the priming effect, the more this brain area is activated. Although we here suggest that, in particular, less empathic participants are prone to the effect, we cannot directly link empathy with somatosensory cortex activity in our experiment.

The present results suggest that empathy is related to social priming. But how does this personality trait affect priming? Our results demonstrate that the neural basis of the “hard on crime” effect is in particular the somatosensory cortex. The “hard on crime” effect describes that incidental tactile experiences influence how “hard” we are in making punishment recommendations. This tactile priming effect seems to rely on somatosensory cortex activity. Empathy may have resulted in higher active attention toward the protagonist in the story and thereby decreased the more passive sensory/somatosensory influence of the tactile priming. Examining the chameleon effect (the non-conscious mimicry of behavior of one’s interaction partners), Chartrand and Bargh demonstrated that the more empathic an individual was, the more he or she imitated others, resulting in stronger bonds with that person (Chartrand and Bargh, 1999). Thus, paying more attention to (verbal or non-verbal) behavior of others may override or reduce other influences on participant’s behavior and brain activation such as tactile priming.

The present results are also in line with behavioral studies reporting intriguing findings for prosocial behavior caused by briefly experienced interpersonal touch. For example, in the so-called Midas touch experiment Crusco and Wetzer demonstrated the social power of touch by examining tipping behavior in a restaurant. Waitresses were instructed to briefly touch the customers on the shoulder or the palm of the hand when they went to get change. Customers who were touched became more generous (Crusco and Wetzel, 1984). This link between touch and prosocial behavior has confirmed by numerous similar studies (Goldman et al., 1985; Stephen, 1986; Hornick, 1992; Lynn et al., 1998). Since prosocial behavior is seen as a key component of empathy, we believe that those studies confirm our hypothesis that touch is linked to empathy. Thus, given that touch may be the first sense we develop in our life, the tactile sense might be much more important for our social perceptions and behavior than previously thought.

For personality factors beyond empathy (Big-Five factors), we did not find any effects (neither with tactile priming effects nor with the related brain responses). Although the neuro-anatomical basis of the Big Five is still discussed, previous imaging studies related neuroticism to prefrontal-temporal regions, extraversion to precuneus and superior temporal cortex areas, openness to prefrontal-parietal brain regions, agreeableness to prefrontal cortex and fusiform gyrus, and conscientiousness to prefrontal areas (Riccelli et al., 2017). Earlier studies examining relationships between empathy and Big-Five personality factors (e.g., (Mooradian et al., 2011; Melchers et al., 2016) showed that for EC agreeableness and for FS and PT openness seem to be important dimensions. As expected, PD was a main predictor for neuroticism. In our study, we did not find any correlations of the Big Five with empathy scores. However, this may be explained by our small sample size.

Given that the classic understanding of the somatosensory cortex is to represent tactile stimulation perceived on our body surface, it may be surprising that we here discuss roles of the somatosensory cortices for priming effects in social judgments. How can the somatosensory cortices on the one hand represent touch in a more or less mechanical way, but on the other hand also be involved in higher cognitions such as social judgments? This may be explained by the theory of neural reuse. Anderson’s theory of neural reuse argues that neural elements originally developed for one purpose are put to multiple uses (Anderson, 2010). In his theory, he argues that the cognitive roles played by each region of the brain are various. Thus, the neural reuse theory refers to a form of neuroplasticity, in which brain areas are involved in different neural partnerships depending on tasks and circumstances.

Several limitations of the outcome of this study have to be mentioned. First, the number of participants is very small. Therefore, the present study should be classified as a pilot study. Future studies should enlarge the number of participants. This is also important because the majority of our participants were females. Since empathy measures are known to be sensitive to gender [e.g., (Schmitt et al., 2008)], future studies with more participants should control this variable. Second, we here used only the IRI as a test for trait empathy. Considering that there are numerous tests for trait empathy and that these tests are based on different concepts of empathy (Neumann et al., 2015), further measures of empathy would be desirable. However, the IRI we used in the present study is widely accepted in neuroimaging contexts. Third, we only tested for trait empathy. It would be interesting if state empathy would result in similar relationships. Last, we do not have any behavioral data to support our assumption that participants emphasized with the protagonists in the scenario. Further studies are needed to examine how empathy interacts with the effect.

The results of the present study suggest that previously shown embodiment effects in the tactile domain (tactile priming of social judgments) can be predicted by empathy personality traits. Thus, we conclude that empathy seems to protect from tactile priming effects in the social domain. The more empathic we are, the less we are prone to unconscious priming effects, such as recommending harder sentences when briefly experiencing something hard before. Therefore, empathic feelings toward someone else may help us to avoid being affected by incident (haptic) factors in the surrounding world.

## Data Availability Statement

The datasets generated for this study are available on request to the corresponding author.

## Ethics Statement

The studies involving human participants were reviewed and approved by Ethics Committee of the University of Magdeburg. The patients/participants provided their written informed consent to participate in this study.

## Author Contributions

LC, HS, SM, AH, AS, MSchaefer, CD, CS, MSchäfer, and JB conceived and designed the experiment. CS contributed analysis tools. NM and MSchaefer collected and analyzed the data. MSchaefer, JB, CD, and AH wrote the manuscript.

## Conflict of Interest

The authors declare that the research was conducted in the absence of any commercial or financial relationships that could be construed as a potential conflict of interest.

## References

[B1] Ackerman J. M. Nocera C. C. Bargh J. A. (2010). Incidental haptic sensations influence social judgments and decisions. *Science* 328 1712–1715. 10.1126/science.1189993 20576894 PMC3005631

[B2] Allen M. Frank D. Glen J. C. Fardo F. Callaghan M. F. Rees G. (2017). Insula and somatosensory cortical myelination and iron markers underlie individual differences in empathy. *Sci. Rep.* 7 43316. 10.1038/srep43316 PMC5335674 28256532

[B3] Anderson M. L. (2010). Neural reuse: a fundamental organizational principle of the brain. *Behav. Brain Sci.* 33 245–266; discussion 266–313. 20964882 10.1017/S0140525X10000853

[B4] Banissy M. J. Kanai R. Walsh V. Rees G. (2012). Inter-individual differences in empathy are reflected in human brain structure. *Neuroimage* 62 2034–2039. 10.1016/j.neuroimage.2012.05.081 22683384 PMC3778747

[B5] Batson C. D. Duncan B. D. Ackerman P. Buckley T. Birch K. (1981). Is empathic emotion a source of altruistic motivation? *J. Pers. Soc. Psychol.* 40 290–302. 10.1037/0022-3514.40.2.290

[B6] Blakemore S. J. Bristow D. Bird G. Frith C. Ward J. (2005). Somatosensory activations during the observation of touch and a case of vision-touch synaesthesia. *Brain* 128(Pt 7) 1571–1583. 10.1093/brain/awh500 15817510

[B7] Blankenburg F. Ruff C. C. Deichmann R. Rees G. Driver J. (2006). The cutaneous rabbit illusion affects human primary sensory cortex somatotopically. *PLoS Biol.* 4:e69. 10.1371/journal.pbio.0040069 16494530 PMC1382015

[B8] Borkenau P. Ostendorf F. (1993). *Neo-Fünf-Faktoren Inventar (NEO-FFI) Nach Costa und McCrae.* Göttingen: Hogrefe.

[B9] Bufalari I. Aprile T. Avenanti A. Di Russo F. Aglioti S. M. (2007). Empathy for pain and touch in the human somatosensory cortex. *Cereb. Cortex* 17 2553–2561. 10.1093/cercor/bhl161 17205974

[B10] Chartrand T. L. Bargh J. A. (1999). The Chameleon effect: the perception-behavior link and social interaction. *J. Perso. Soc. Psychol.* 76 893–910. 10.1037/0022-3514.76.6.893 10402679

[B11] Costa P. T. McCrae R. R. (1992). *Revised NEO Personality Inventory (NEO-PI-R) and NEO Five-Factor Inventory (NEO-FFI) Professional Manual.* Odessa, FL: Psychological Assessment Resources.

[B12] Crusco A. H. Wetzel C. G. (1984). The midas touch. The effecs of interpersonal touch on restaurant tipping. *Perso. Soc. Psychol. Bull.* 10 512–517. 10.1177/0146167284104003

[B13] Davis M. H. (1983). Measuring individual differences in empathy: evidence for a multidimensional approach. *J. Pers. Soc. Psychol.* 44 113–126. 10.1037/0022-3514.44.1.113

[B14] De Waal F. B. M. Preston S. D. (2017). Mammalian empathy: behavioural manifestations and neural basis. *Nat. Rev. Neurosci.* 18 498–509. 10.1038/nrn.2017.72 28655877

[B15] Denke C. Rotte M. Heinze H. J. Schaefer M. (2016). Lying and the subsequent desire for toothpaste: activity in the somatosensory cortex predicts embodiment of the moral-purity metaphor. *Cereb Cortex* 26 477–484. 25214311 10.1093/cercor/bhu170

[B16] Gallese V. Lakoff G. (2005). The Brain’s concepts: the role of the Sensory-motor system in conceptual knowledge. *Cogn. Neuropsychol.* 22 455–479. 10.1080/02643290442000310 21038261

[B17] Gallo S. Paracampo R. Muller-Pinzler L. Severo M. C. Blomer L. Fernandes-Henriques C. (2018). The causal role of the somatosensory cortex in prosocial behaviour. *eLife* 7:e32740. 10.7554/eLife.32740 PMC5973831 29735015

[B18] Gazzola V. Aziz-Zadeh L. Keysers C. (2006). Empathy and the somatotopic auditory mirror system in humans. *Curr. Biol.* 16 1824–1829. 10.1016/j.cub.2006.07.072 16979560

[B19] Goldman M. Kiyohara O. Pfannensteil D. (1985). Interpersonal touch, social labeling and the foot-in-the-door effect. *J. Soc. Psychol.* 152 143–147. 10.1080/00224545.1985.9922866

[B20] Hoffman M. L. (2007). “Empahty, its arousal, and prosocial functioning,” in *Emapthy and Moral Development: Implications for Caring and Justice*, ed. Hoffman M. L. (New York, NY: Cambridge University Press).

[B21] Hooker C. I. Verosky S. C. Germine L. T. Knight R. T. D’Esposito M. (2010). Neural activity during social signal perception correlates with self-reported empathy. *Brain Res.* 1308 100–113. 10.1016/j.brainres.2009.10.006 19836364 PMC2798147

[B22] Hornick J. (1992). Tactile stimulation and consumer response. *J. Consum. Res.* 19 449–458.

[B23] Keysers C. Kaas J. H. Gazzola V. (2010). Somatosensation in social perception. *Nat. Rev. Neurosci.* 11 417–428. 10.1038/nrn2833 20445542

[B24] Keysers C. Wicker B. Gazzola V. Anton J. L. Fogassi L. Gallese V. (2004). A touching sight: SII/PV activation during the observation and experience of touch. *Neuron* 42 335–346. 15091347 10.1016/s0896-6273(04)00156-4

[B25] Khan S. Michmizos K. Tommerdahl M. Ganesan S. Kitzbichler M. G. Kenet T. (2015). Somatosensory cortex functional connectivity abnormalities in autism show opposite trends, depending on direction and spatial scale. *Brain* 138 1394–1409. 10.1093/brain/awv043 25765326 PMC5013931

[B26] Lacey S. Stilla R. Sathian K. (2012). Metaphorically feeling: comprehending textural metaphors activates somatosensory cortex. *Brain Lang.* 120 416–421. 10.1016/j.bandl.2011.12.016 22305051 PMC3318916

[B27] Lakoff G. Johnson M. (1980). *Metaphors we Live.* Chicago, IL: University of Chicago Press.

[B28] Lynn M. Le J.-M. Sherwyn D. (1998). Reach out and touch your customers. *Corn. Hotel Restaur. Admin. Q.* 39 60–65. 10.1016/s0010-8804(98)80298-x

[B29] Melchers M. C. Li M. Haas B. W. Reuter M. Bischoff L. Montag C. (2016). Similar personality patterns are associated with empathy in four different countries. *Front. Psychol.* 7:290. 10.3389/fpsyg.2016.00290 PMC4781828 27014115

[B30] Montag C. Gallinat J. Heinz A. (2008). Theodor Lipps and the concept of empathy: 1851-1914. *Am. J. Psychiatry* 165:1261. 10.1176/appi.ajp.2008.07081283 18829882

[B31] Mooradian T. A. Davis M. Matzler K. (2011). Dispositional empathy and the hierarchical structure of personality. *Am. J. Psychol.* 124 99–109. 21506454 10.5406/amerjpsyc.124.1.0099

[B32] Neumann D. L. Chan R. C. K. Boyle G. J. Wang Y. Westbury H. R. (2015). “Measures of empathy: self-report, behavioral, and neuroscientfic approaches,” in *Measures of Personality and Social Psychology Constructs*, eds Boyle G. J. Saklofske D. H. (Amsterdam: Elsevier).

[B33] Paulus C. (2009). *Der Saarbrücker Persönlichkeitsfragebogen SPF(IRI) zur Messung von Empathie: Psychometrische Evaluation der deutschen Version des Interpersonal Reactivity Index.* Saarbrücken: Universität des Saarlandes.

[B34] Preston S. D. (2013). The origins of altruism in offspring care. *Psychol. Bull.* 139 1305–1341. 10.1037/a0031755 23458432

[B35] Preston S. D. De Waal F. B. (2002). Empathy: Its ultimate and proximate bases. *Behav. Brain Sci.* 25 1–20; discussion 20-71. 12625087 10.1017/s0140525x02000018

[B36] Riccelli R. Toschi N. Nigro S. Terracciano A. Passamonti L. (2017). Surface-based morphometry reveals the neuroanatomical basis of the five-factor model of personality. *Soc. Cogn. Affect. Neurosci.* 12 671–684. 28122961 10.1093/scan/nsw175 PMC5390726

[B37] Ruby P. Decety J. (2004). How would you feel versus how do you think she would feel? A neuroimaging study of perspective-taking with social emotions. *J. Cogn. Neurosci.* 16 988–999. 10.1162/0898929041502661 15298786

[B38] Schaefer M. Cherkasskiy L. Denke C. Spies C. Song H. Malahy S. (2018). Incidental haptic sensations influence judgment of crimes. *Sci. Rep.* 8:6039. 10.1038/s41598-018-23586-x PMC5902547 29662068

[B39] Schaefer M. Denke C. Heinze H. J. Rotte M. (2014). Rough primes and rough conversations: evidence for a modality-specific basis to mental metaphors. *Soc. Cogn. Affect. Neurosci.* 9 1653–1659. 10.1093/scan/nst163 24097375 PMC4221208

[B40] Schaefer M. Flor H. Heinze H. J. Rotte M. (2006). Dynamic modulation of the primary somatosensory cortex during seeing and feeling a touched hand. *Neuroimage* 29 587–592. 10.1016/j.neuroimage.2005.07.016 16099177

[B41] Schaefer M. Flor H. Heinze H. J. Rotte M. (2007). Morphing the body: illusory feeling of an elongated arm affects somatosensory homunculus. *Neuroimage* 36 700–705. 10.1016/j.neuroimage.2007.03.046 17499523

[B42] Schaefer M. Heinze H. J. Rotte M. (2012). Embodied empathy for tactile events: Interindividual differences and vicarious somatosensory responses during touch observation. *Neuroimage* 60 952–957. 10.1016/j.neuroimage.2012.01.112 22306799

[B43] Schaefer M. Rotte M. Heinze H. J. Denke C. (2013). Mirror-like brain responses to observed touch and personality dimensions. *Front. Hum. Neurosci.* 7:227. 10.3389/fnhum.2013.00227 PMC3665908 23754999

[B44] Schaefer M. Rotte M. Heinze H. J. Denke C. (2015). Dirty deeds and dirty bodies: Embodiment of the Macbeth effect is mapped topographically onto the somatosensory cortex. *Sci. Rep.* 5:18051. 10.1038/srep18051 PMC4685311 26686599

[B45] Schaefer M. Xu B. Flor H. Cohen L. G. (2009). Effects of different viewing perspectives on somatosensory activations during observation of touch. *Hum. Brain Mapp.* 30 2722–2730. 10.1002/hbm.20701 19172650 PMC6870795

[B46] Schmitt D. P. Realo A. Voracek M. Allik J. (2008). Why can’t a man be more like a woman? Sex differences in Big Five personality traits across 55 cultures. *J. Pers. Soc. Psychol.* 94 168–182. 10.1037/0022-3514.94.1.168 18179326

[B47] Singer T. Seymour B. O’Doherty J. Kaube H. Dolan R. J. Frith C. D. (2004). Empathy for pain involves the affective but not sensory components of pain. *Science* 303 1157–1162. 10.1126/science.1093535 14976305

[B48] Stephen R. Z. R. (1986). The effect on tipping of a waitress touching male and female customers. *J. Soc. Psychol.* 126 141–142. 10.1080/00224545.1986.9713586

[B49] Xie J. Lu Z. Wang R. Cai Z. G. (2016). Remember hard but think softly: metaphorical effects of hardness/softness on cognitive functions. *Front. Psychol.* 7:1343. 10.3389/fpsyg.2016.01343 PMC5018472 27672373

